# Miscellany

**Published:** 1895-11

**Authors:** 


### Miscellany.

### CRAIG COLONY FOR EPILEPTICS.

The object of a colony for epileptics is to provide for the four great needs of these unfortunates: first, to give them schools where they may be educated as other children and young people are; second, to afford them industrial training in any sort of occupation they may desire to follow; third, to provide those epileptics a home to whom all other doors are closed; fourth, to treat every case of epilepsy according to the best known scientific methods.

The need for provision of this kind is apparent, when we consider that there are 120,000 epileptics in the United States. There are some 12,000 in the state of New York, of whom more than 1,000 are in almshouses and asylums on public charge.

### THE SITUATION OF CRAIG COLONY.

Craig colony, named for the late Oscar Craig, of Rochester, formerly president of the State Board of Charities, consists of nearly 1,900 acres of land in the Genesee Valley. It is reached by two trunk lines of railways (the Erie and the Delaware & Lackawanna) and from roads centering at Rochester by the Western New York & Pennsylvania railroad. The colony has its own post-office and railway station known as Sonyea (Livingston Co.), an Indian word signifying sunny place.

### THE PLAN OF THE COLONY.

The law establishing the colony required that it should be arranged on the village plan. To this end the services of Frederick Law Olmstead, the landscape architect, were secured by the board of managers and he has prepared the scheme of an industrial and agricultural village upon the best principles. There are already many buildings upon the grounds (some thirty or forty) which are to be immediately utilised. Craig colony will not resemble an institution in any particular, but will look more like a country town than anything else. As the patients are received, they will be set to work or at study in various ways. They will take care of the farms, gardens and orchards; they will plan and build new houses. There will be among them, tailors, shoemakers, printers, bookbinders, masons, ironworkers, carpenters, painters and so on. In fact, every sort of employment, every sort of recreation, everything in short that goes to make up the life of a country village will be found in this colony, the only difference being that the citizens of this community will be epileptics.

### OPENING OF THE COLONY.

Work has been progressing very rapidly during the year to prepare existing buildings for the reception of patients. The first quota of patients, numbering sixty, will be taken from the almshouses early in November. It is proposed to receive 200 during the winter and perhaps more. Estimating the capacity of the present buildings at 300, additional buildings will be needed during the coming year to accommodate 300 more patients, before the 600 now in the almshouses can be cared for.

### STATE PATIENTS.

The patients taken from the almshouses and asylums will be known as state patients, and they will be provided for before any private patients can be received. They will be sent to the colony by the poor authorities of each county according to a form required by law, the blanks for which will be furnished on application to the State Board of Charities or the superintendent of the colony.

### PRIVATE PATIENTS.

As soon as all epileptics now upon public charge eligible for admission to the colony are provided for, private patients will be received at prices to be regulated by the board of managers, according to the kind and extent of care and attention required. Such patients may, if it be desired, erect cottages for their own use upon the grounds, upon application to the board of managers.

RESTRICTIONS AS TO THE KIND OF CASES RECEIVED.

There will be no restriction as to the age of patients admitted, and the only restriction practically applies to the mental condition. Insane epileptics, or epileptics subject to insane outbreaks, cannot be taken into the colony.

DONATIONS TO THE COLONY.

The law permits the board of managers to take and hold in trust for the state any grant of land, gift or bequest of money, or any donation to be applied, principal or income, or both, to the maintenance and education of epileptics and the general uses of the colony. Charitably disposed people have here an opportunity for the beneficent use of money, and it is hoped that memorial buildings in the way of chapel, library, museum, gymnasium, school, shop, or cottage houses, bearing the donors' names, may in time be erected.

OFFICERS OF CRAIG COLONY.

The State Board of Charities has jurisdiction over this colony. The board of managers consists of Dr. Frederick Peterson, president, 60 West Fiftieth street, New York ; Mrs. Charles F. Wadsworth, Geneseo ; H. E. Brown, Mount Morris, secretary ; W. H. Cuddeback, Buffalo ; Charles E. Jones, M. D., Albany ; L. S. Oatman, Buffalo ; Judge O. P. Hurd, Watkins ; Jeanette B. Hawkins, Malone, and H. A. Phillips, Lowville. The medical superintendent is Dr. William P. Spratling, Craig Colony, Sonyea, N. Y.

---

THE Buffalo Antitoxic Company, 35 West Eagle street, announces that it is now prepared to furnish a reliable serum for the treatment of diphtheria. The serum has been thoroughly tested and will be found as labeled. Tests have also been made by Dr. Wm. H. Park, of the New York Board of Health, and he reports the serum to be of standard strength.

The production of antitoxin was commenced last December. The plant has been enlarged and perfected and now the finished product is submitted for consideration. During the past few months many physicians have used the antitoxin manufactured by the Buffalo Antitoxic Company in the treatment of diphtheria with good results.

The *antitoxic serum* is supplied in *two strengths*, each vial containing 10 c. c.: No. 1 vial (white label), 800 normal units ; No. 2 vial (red label), 1,500 normal units.

The contents of vial No. 1 will ordinarily suffice for a fully developed case of diphtheria in a child under ten years of age. For a fully developed case of diphtheria in an adult the contents of vial No. 2 should be given.

As a prophylactic for children (who have been exposed to diphtheria), it is advised that ½ c. c. of contents of vial No. 1 be given for each year of their age. For adults, use the entire contents, 10 c. c.

*Administration.*—It is advised that a syringe holding the full quantity be used and injected under the skin at one operation. The place selected should be where large folds of skin can be raised, as the chest, just below the clavicle or thigh. Places on which the patient is accustomed to lie should be avoided.

*Caution.*—The vials which contain the remedial serum should be excluded from light and kept in a cool place. Also, under all circumstances, the general laws of antisepsis should be observed regarding the parts to be injected, and especially the cleansing of the *injector* and *needle.*

*Remarks.*—The horses selected by the Buffalo Antitoxic Company for the production of antitoxin are young, healthy and absolutely sound. The serum is prepared with the most scrupulous care. During the immunising period the temperatures are taken twice daily, thereby showing the progress. The utmost care in food, grooming and exercise is observed, thus insuring a healthy condition of the animal.

---

RECENT graduate or student wanted to devote four or five hours a day to visiting local physicians in the interest of our specialties: Ferratin, Lactophenin, Papain, Cocaine, etc. Address C. F. Boehringer & Soehne, 7 Cedar street, New York.

